# The Impact of Polycyclic Aromatic Hydrocarbons Exposure from Seasonal Biomass Burning on Oxidative Stress and Genotoxicity Biomarkers in Patients with Diabetes and Hypertension in Chiang Mai, Thailand

**DOI:** 10.3390/toxics14090771

**Published:** 2026-08-29

**Authors:** Muhammad Ali, Xianfeng Cao, Udomsap Jaitham, Peerapong Jeeno, Anurak Wongta, Saweang Kawichai, Sumed Yadoung, Surat Hongsibsong

**Affiliations:** 1 School of Health Sciences Research, Research Institute for Health Sciences, Chiang Mai University, Chiang Mai 50200, Thailand; muhammad_ali@cmu.ac.th (M.A.); udomsap_j@cmu.ac.th (U.J.); peerapong_jeen@cmu.ac.th (P.J.); anurak.wongta@cmu.ac.th (A.W.); sumed.yadoung@cmu.ac.th (S.Y.); 2 Environmental, Occupational Health Sciences and Non-Communicable Diseases Center of Excellence, Research Institute for Health Sciences, Chiang Mai University, Chiang Mai 50200, Thailand; xianfeng_cao@cmu.ac.th (X.C.); sawaeng.kaw@cmu.ac.th (S.K.)

**Keywords:** polycyclic aromatic hydrocarbons, lipid peroxidation, DNA damage, oxidative stress, diabetes, hypertension

## Abstract

Polycyclic aromatic hydrocarbons (PAHs) are produced primarily by incomplete combustion of organic materials, including biomass burning, vehicle exhaust, and forest fires. Exposure to PAHs can induce reactive oxygen species (ROS) and oxidative stress, leading to lipid peroxidation and DNA damage. Biomass burning is a major environmental and public health problem in Chiang Mai province, containing particulate matter linked with respiratory and cardiovascular diseases. This prospective observational study aimed to evaluate the association between oxidative stress biomarkers and PAH exposure. A total of 152 participants comprised healthy controls (88), diabetes patients (35), hypertension patients (15) and diabetes + hypertension patients (14). Only participants aged more than 18 years were included. Participants who had acute respiratory infection or malignancies were excluded. This study was conducted in the pre-burning (December) and post-burning (May) periods by measuring biomarkers such as 1-OHP, MDA and 8-OHdG. Urinary 1-OHP increased significantly in the post-burning period from 0.21 ± 0.50 to 0.87 ± 1.25 μmol/mol creatinine (*p* < 0.001). Serum 8-OHdG increased significantly in all participants from 51.04 ± 24.90 to 66.08 ± 27.82 pg/mL (*p* < 0.001) and increased significantly in the diabetic and hypertension group. Serum MDA did not show any significant change post-burning (6.09 ± 3.71 to 6.00 ± 3.71 nmol/mL; *p* = 0.825), but MDA significantly decreased in the diabetic group (*p* = 0.017). 8-OHdG tended to increase in all clinical groups in the post-burning season compared to the pre-burning season, indicating oxidative DNA damage. 8-OHdG was found to be a sensitive biomarker of oxidative stress, suggesting its potential as an early indicator in haze season in vulnerable populations.

## 1. Introduction

Air pollution, particularly particulate matter and polycyclic aromatic hydrocarbons, poses a major health risk in Southeast Asia, especially in northern Thailand [1,2,3]. Seasonal biomass burning and anthropogenic emissions contribute to severe air pollution in Southeast Asia, particularly in Chiang Mai, where PM2.5 levels often exceed national and international guidelines [4,5,6]. On an annual basis, more than 7 million individuals succumb to premature mortality because of air pollution, surpassing all other categories of pollution [7].

Polycyclic aromatic hydrocarbons (PAHs) from vehicular emissions, industrial activities, biomass burning, and other combustion sources can penetrate the lungs and circulation and cause inflammation, increasing the risk of respiratory, cardiovascular, metabolic, and neurodegenerative diseases [8]. Extended exposure to polluted air has been systematically correlated with heightened morbidity and mortality rates, especially in relation to chronic non-communicable diseases [9].

Enzymatic bioactivation of polycyclic aromatic hydrocarbons results in the formation of redox-active metabolites that further elevate oxidative stress [10]. Polycyclic aromatic hydrocarbons are metabolically activated by the cytochrome P450 enzyme, which catalyzes the process of conversion into reactive intermediates such as quinones and epoxides [11]. Oxidative stress shows a dysregulated redox equilibrium. Oxidative stress is detected by the excessive generation and accumulation of reactive oxygen species (ROS), alongside the impairment of antioxidant systems within cells or tissues [12,13].

Polycyclic aromatic hydrocarbons (PAHs) have been demonstrated to generate reactive oxygen species (ROS) via the redox cycle, which has the potential to provoke oxidative alterations of deoxyribonucleic acid (DNA) and lipids in vivo [14]. PM2.5-mediated oxidative stress precipitates damage to lipids and DNA, phenomena that are pivotal to vascular impairment and metabolic dysregulation. In the context of hypertension, augmented lipid peroxidation may play a significant role in endothelial damage and the rigidity of arteries, whereas in diabetes, oxidative injury to DNA could potentially intensify insulin resistance and the dysfunction of pancreatic β-cells [15]. Diabetes mellitus (DM) is one of the biggest threats to worldwide public health [12]. Diabetes mellitus (DM) is a chronic disease, characterized by hyperglycemia, which happens due to the inability of the body to either produce insulin (Type 1 DM or T1DM) or utilize insulin effectively (Type 2 DM or T2DM) [16]. In individuals diagnosed with diabetes mellitus, vascular endothelial cells are frequently subjected to damage induced by oxidative stress, which compromises endothelial junctions, enhances intravascular permeability, and ultimately plays a significant role in the pathogenesis of both macrovascular and microvascular complications [12]. Endothelial dysfunction is characterized as a systemic, insidious, and reversible pathological condition affecting the endothelium, resulting from diminished nitric oxide bioavailability and compromised vasodilatory function, which is concomitantly linked with a pro-inflammatory and prothrombotic status [17].

**Figure 1:**
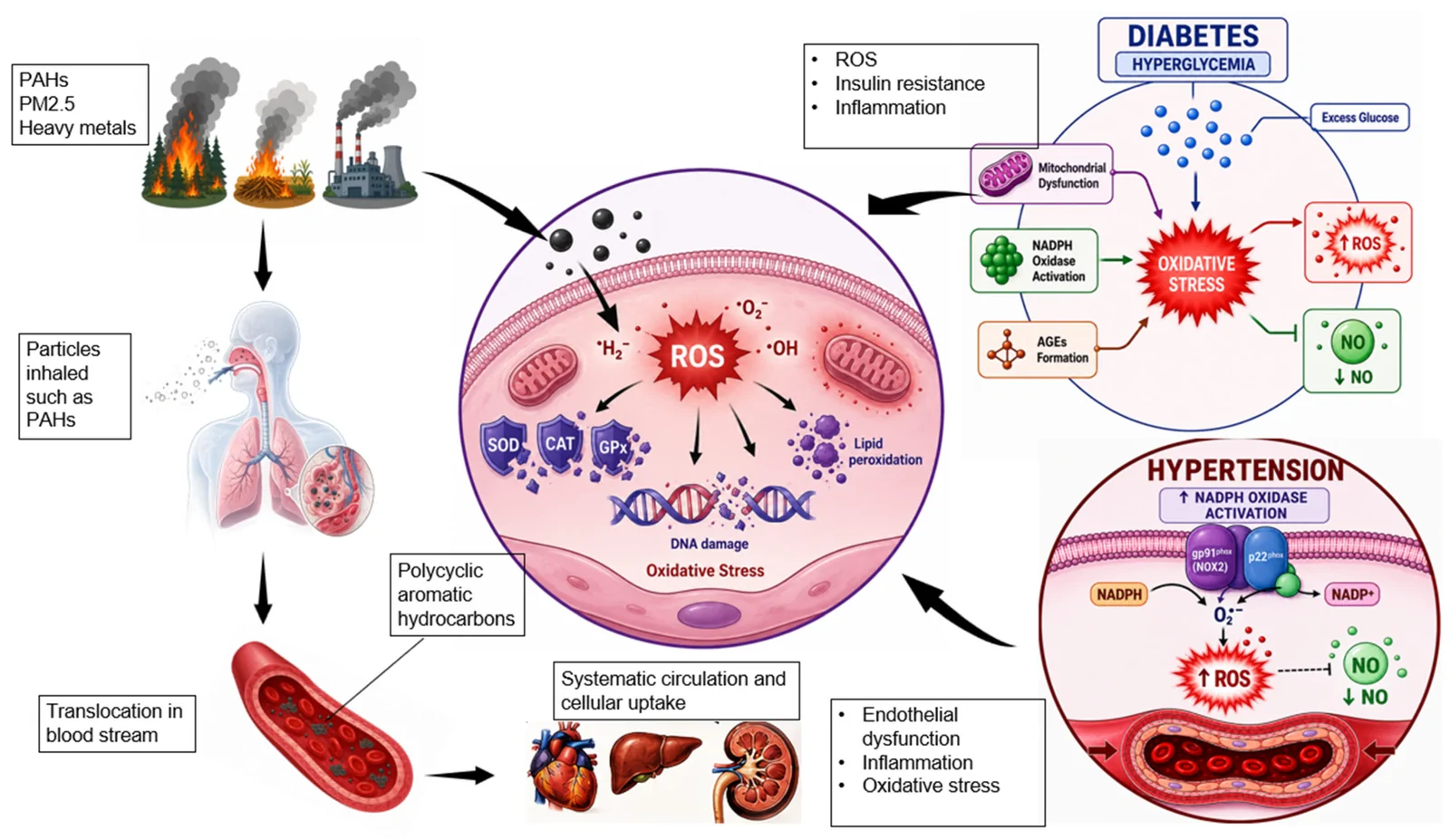
Schematic representation of air pollution exposure, inducing systemic oxidative stress and causing lipid peroxidation and DNA damage, and its association with diabetes and hypertension. (Abbreviations: AGEs: advanced glycation end products; SOD: superoxide dismutase; CAT: catalase; GPx: glutathione peroxidase; NADPH: nicotinamide adenine dinucleotide phosphate; CYP: cytochrome P450).

Figure 1 illustrates the proposed biological mechanism underlying the present study. During the biomass burning season, increased exposure to PM2.5 and PAHs promotes the generation of reactive oxygen species, leading to oxidative DNA damage and lipid peroxidation. The oxidation of DNA predominantly affects guanine residues, attributable to their relatively low oxidation potential. Among reactive oxygen species, the hydroxyl radical exhibits the highest reactivity and specifically targets the C8 position of deoxyguanosine within the DNA structure. This biochemical interaction results in the synthesis of 8-oxo-7,8-dihydro-2-deoxyguanosine (8-oxo-dG), which is frequently quantified as 8-OHdG in various biological matrices. Diabetes and hypertension are already associated with increased oxidative stress; individuals with these conditions may be more susceptible to additional oxidative damage following air pollution exposure. Based on this mechanism, we hypothesized that PAH exposure during the burning season would be associated with increased urinary 1-OHP and elevated oxidative stress biomarkers, particularly 8-OHdG and MDA. It is apparent that biomarkers indicative of oxidative stress are elevated in conditions of systemic oxidative stress, correlating with the severity of hypertension and complications associated with diabetes mellitus [18].

Limited information is available on seasonal changes in PAH exposure and oxidative stress biomarkers among patients with diabetes and hypertension in Chiang Mai during the biomass-burning season. Given the different levels of air pollution before and after the burning season in Chiang Mai, it is necessary to observe the oxidative stress caused by air pollution in vulnerable populations such as diabetic and hypertensive patients in comparison to a healthy control group by measuring oxidative stress biomarkers (MDA and 8-OHdG) and measuring the PAH exposure metabolite (1-OHP). This study covered three different locations with different biomass-burning exposures to observe biomarker levels. Children are also included in the vulnerable population, but our focus is on diabetic and hypertensive patients. The aim of this study was to evaluate the effect of seasonal biomass-burning-related PAH exposure on oxidative stress and oxidative DNA damage by measuring urinary 1-OHP, serum malondialdehyde (MDA), and 8-hydroxy-2′-deoxyguanosine (8-OHdG) in healthy individuals and patients with diabetes, hypertension, or both in Chiang Mai, Thailand. This study also compared these biomarkers between the low- and high-burning seasons and among the different participant groups.

## 2. Materials and Methods

### 2.1. Study Design and Population

**Figure 2:**
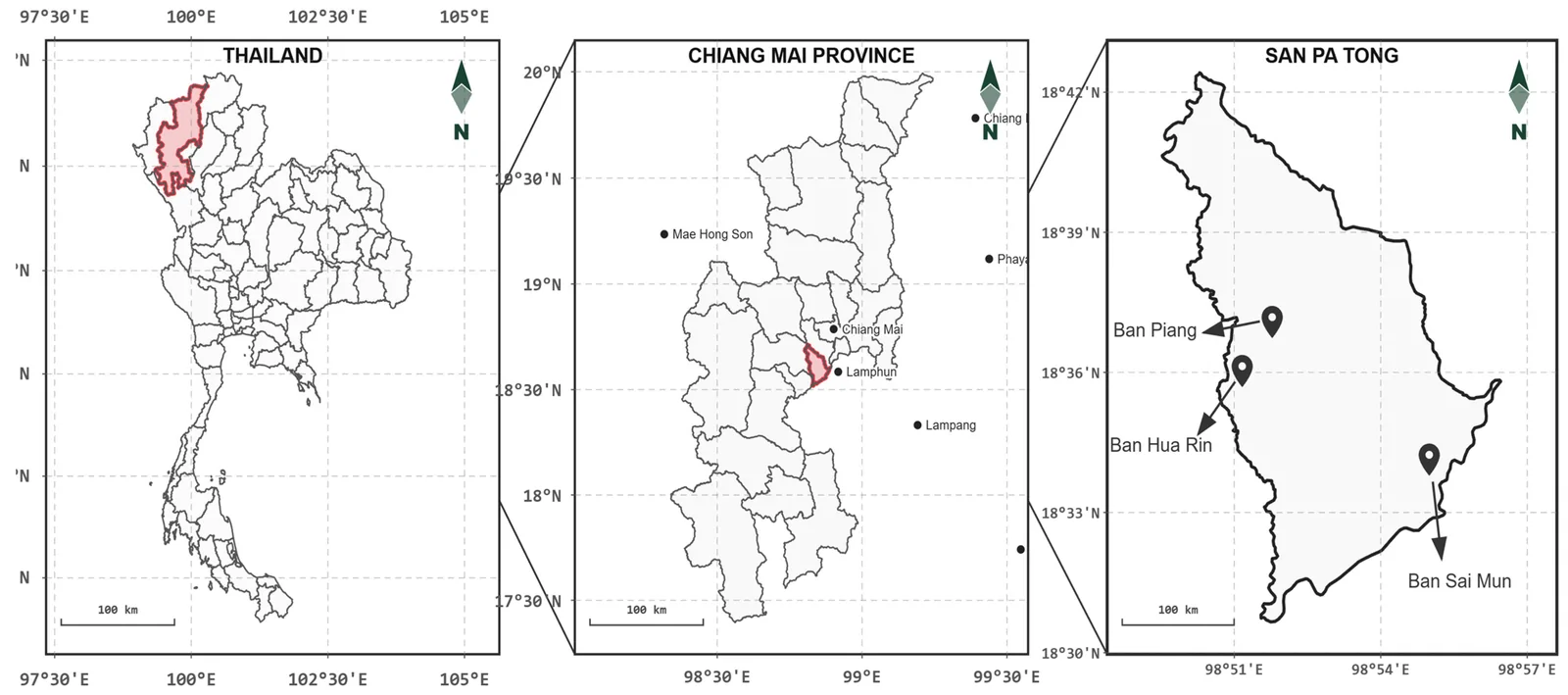
Study area and sampling locations. San Pa Tong district (Chiang Mai province) with the three sampling communities (Ban Hua Rin, Ban Piang and Ban Sai Mun) indicated by black markers.

This investigation constituted a prospective observational study conducted in Ban Hua Rin, Thung Satok Subdistrict (Site 1); Ban Piang, Ban Mae Subdistrict (Site 2); and Ban Sai Mun, Mae Ka Subdistrict (Site 3), located within the San Pa Tong District of Chiang Mai province, as shown in Figure 2, with data collection occurring in December 2023 for the first visit, which assessed pre-burning air pollution, and in May 2024 for the second visit, which evaluated post-burning air pollution. In these areas, there was significant biomass burning from January to April, so we took samples in December that were considered pre-burning and took samples during the second visit in May that were also considered to be from the post-burning season. These sites were selected because of extensive biomass burning, agricultural residue burning, forest fires and meteorological conditions and based on pre-existing air pollution data indicating differences in combustion-related pollution and seasonal PM2.5 exposure too. Participants were enrolled as a longitudinal study cohort, and biological samples were collected from the same individuals two times before and after the biomass-burning season.

A total of 153 participants were recruited, with 152 participants involved in both visits. Participants who were aged more than 18 years and resided in that area for at least six months were included. Individuals with acute respiratory infections, known malignancies, or current corticosteroid use were also excluded. Participants were tested for blood glucose levels, waist circumference, blood cholesterol and blood pressure. Participants with normal measurements and no previous history of diabetes or hypertension were enlisted in a healthy control group. Participants who were diagnosed with diabetes mellitus and hypertension were enlisted from community health facilities in Chiang Mai. Participants who were diagnosed with diabetes mellitus and hypertension were enlisted from community health facilities in Chiang Mai. The criterion for choosing diabetic patients was a fasting glucose value of more than 126 mg/dL; the criterion for choosing patients with hypertension was systolic and diastolic blood pressure of more than 140/90. Participants were counseled to refrain from doing heavy physical exercise, consuming alcoholic beverages, and ingesting foods that are high in antioxidants for 12 h preceding the collection of blood and urine samples. Individuals exhibiting poorly managed diabetes (operationally defined as HbA1c levels exceeding 10%) or those presenting with acute respiratory infections at the time of enrollment were systematically excluded from the study to minimize potential bias.

### 2.2. Ethical Approval and Informed Consent

This research involving human subjects received authorization from the Ethical Committee of the Associate Medical Sciences at Chiang Mai University (protocol code AMSEC-66EX-062). The investigations were carried out in compliance with local regulations and institutional stipulations. The individuals involved in the study provided their written informed consent to engage in this research.

### 2.3. Data Collection

Samples and data were collected from a project entitled “Assessment of NCDs indicators in PM2.5 exposure individuals to develop screening innovation system Telehealth for sustainable community”. The design of the parent study was prospective, longitudinal, and community-based, with feasibility evaluation of a telehealth-based NCD screening system. Inclusion criteria were participants aged more than 18 years who could give informed consent and resided in that area for at least 6 months, with no acute respiratory disease. Participants who were diagnosed with diabetes and hypertension were included in the study. Exclusion criteria included participants with other chronic diseases, such as malignancies. Participants who were not willing to participate in both sample collection periods were excluded. Variables that were collected included demographics (age, sex, education, occupation, income, residence duration, marital status), lifestyle (smoking, drinking, physical exercise frequency, sleep duration), covariates related to NCDs (blood pressure, glucose, total cholesterol, waist circumference, BMI, previous diagnosis of hypertension or diabetes, family history of diabetes or cardiovascular diseases and cancer, medication use for chronic diseases, symptoms related to eye, skin, respiratory, fainting), and a questionnaire related to KAB (knowledge, attributes, behavior). The sample collection protocol for urine was to collect early-morning urine samples, and blood samples were collected after 8–12 h fasting to determine glucose. These samples were then transported on ice; after centrifugation, serum was stored at −80 °C until analysis.

Venous blood (3 mL) samples of healthy participants and patients with diabetes and hypertension were collected by expert laboratory technicians. Early-morning urine samples of all participants were also collected in sterile polyethylene containers. Both blood and urine samples were kept on ice to be transported to the Research Institute for Health Science, Chiang Mai University. A 3000× *g* centrifuge machine was used to separate serum from blood at 4 °C for 15 min. After separation of serum, the serum was stored at −80 °C until further analysis.

### 2.4. Variables and Covariates

There were many variables that were measured at the time of data collection, such as waist circumference, total cholesterol, systolic/diastolic blood pressure, glucose, alcohol drinking and exercise frequency. The variable-monitoring equipment included an electronic blood pressure monitor (Yuwell YE670D; Jiangsu Yuyue Medical Equipment & Supply Co., Ltd., Danyang, Jiangsu, China) and a waist measurement tape; participants were asked about the frequency of alcoholic beverage consumption, with responses categorized as 0 = never, 1 = once a month, 2 = 2–4 times per month, 3 = 2–3 times per week, 4 = four or more times per week, and 5 = daily consumption. Blood glucose was measured by a POCT (Point-of-Care Testing) device (ACCU-CHEK^®^ Instant; Roche Diagnostics; Rotkreuz, Switzerland), and total cholesterol was measured by a lipid analyzer (Accutrend^®^ Plus; Roche Diagnostic GmbH, Mannheim, Germany).

### 2.5. PM2.5 Measurements

The atmospheric quality assessment stations within the designated research area were situated across three specific locales of San Pa Tong District, Chiang Mai province; Ban Hua Rin in the Thung Satok Subdistrict; Ban Piang, located in the Ban Mae Subdistrict; and Ban Sai Mun, found in the Mae Ka Subdistrict. The monthly atmospheric quality pertaining to PM2.5, which was quantified from September 2023 through May 2024, was obtained and systematically measured and documented using the Northern Thailand Air Quality Health Index (NTAQHI) [19] (https://www.ntaqhi.info, accessed on 26 August 2026), which is an academic and community-based air quality monitoring network established and maintained by the Research Institute for Health Sciences (RIHES), Chiang Mai University. Low-cost particulate sensors were deployed across the study sites as part of an institutional research framework to provide high-spatial-resolution real-time tracking during the biomass-burning season. Regarding calibration and validation, the NTAQHI sensor nodes are routinely maintained, serviced, and quality-assured under the oversight of Chiang Mai University researchers to ensure reliable data consistency and performance against reference standards. The NTAQHI system continuously measures real-time particulate metrics (PM2.5, PM10, and PM1.0) alongside meteorological parameters (temperature and relative humidity). The index categorizes air quality based on 24 h average PM2.5 concentrations utilizing health-based breakpoints and advisory thresholds adapted from the US EPA guidelines to effectively communicate health risks to vulnerable populations.

### 2.6. 1-Hydroxy Pyrene (1-OHP) Measurements

Based on a previous study [20], urinary 1-hydroxypyrene (1-OHP) was measured using high-performance liquid chromatography, and creatinine was measured using the Jaffe method. The values of 1-OHP were expressed as µmol/mol creatinine. The quality control procedures included certified reference materials, blank samples, duplicate analyses (10% of total samples), and calibration curves exhibiting R^2^ values ≥ 0.99. In the enzymatic hydrolysis step, a 10 mL urine sample was adjusted to pH 5.0 using 1M HCl and transferred into a 50 mL screw-cap test tube containing 10 mL of 0.1M acetate buffer and 25 µL of β-glucuronidase from Helix pomatia. The mixture was vortexed for 10 s and incubated at 37 °C for 2 h.

In a Solid-Phase Extraction (SPE) procedure, samples were vacuum-eluted using an SPE VertiPak C18 3 mL cartridge, and pre-conditioned with 1 mL of methanol (three times) and 1 mL of water (three times). After loading, the cartridge was washed with 10 mL of water and 10 mL of 20% methanol, left to stand for approximately 10 min, and eluted with 10 mL of methanol. The eluate was evaporated to dryness under a stream of nitrogen gas, reconstituted in 200 µL of methanol, and filtered through a 0.2 µm PTFE syringe filter.

Under HPLC-FLD Instrumentation and Conditions, analysis was performed using an Agilent 1260 Infinity II HPLC system (Agilent Technologies, Santa Clara, CA, USA) with a 20 µL injection volume. The mobile phase consisted of 45% water (Line A) and 55% acetonitrile (Line B) at a flow rate of 0.80 mL/min. Separation was achieved using an Infinity Lab Poroshell 120 EC-C18 column; Agilent Technologies, Santa Clara, CA, USA) (25 cm × 5 mm × 5 μm) maintained at 25 °C. Detection was carried out using a fluorescence detector (FLD, 1260 Infinity II, Agilent, CA, USA) with an excitation wavelength of 242 nm, an emission wavelength of 388 nm, PMT gain set to 10, and a response time of 0.25 s, with a total run time of 20 min.

During the method validation step, the limits of quantification (LOQ) and limits of detection (LOD) for the assay were 0.7140 ng/mL and 0.0430 ng/mL, respectively.

### 2.7. Serum Malondialdehyde (MDA) Assessment

The thiobarbituric acid reactive substance (TBARS) assay was the analytical technique employed for the detection of lipid oxidation. This assay is specifically designed to quantify malondialdehyde (MDA), which is one of the terminal products formed during the decomposition of lipid peroxidation derivatives. Serum concentrations of MDA were ascertained utilizing a modified approach based on the methodology outlined by [21]. To perform the assay, a 0.2 mL volume of the sample was combined with 0.2 mL of 0.2M H_3_PO_4_. The color reaction was initiated by adding 25 µL of a 0.11 M thiobarbituric acid solution. The mixture was then heated at 90 °C for 50 min using a water bath. After cooling, the TBARSs (resulting in a pink complex color) were extracted by adding 0.4 mL of n-butanol. Centrifugation at 6000× *g* for 10 min allowed for the separation of the butanolic phase. Each sample was transferred to a 96-well plate and read at 535 nm using a microplate reader (CLARIOstar ^plus^; BMG LABTECH, Ortenberg, Germany). A calibration curve was prepared using MDA standards. The serum concentration of MDA was expressed in nmol per mL. To minimize potential interferences from compounds that react or absorb at 535 nm, each sample was accompanied by a blank tube (sample without the TBA reagent), and the absorbance of the blank tube was subtracted from each sample measurement [22]. In addition, the use of butanol as the stripping agent for the TBARS complex helped to mitigate many of these interferences.

### 2.8. Serum 8-Hydroxy-2-deoxyguanosine (8-OHdG) Assessment

8-OHdG was determined by enzyme-linked immunosorbent assay [23], using a commercial ELISA kit. The standard curve ranged from 0 to 320 pg/mL, the sample dilution factor was 1:5, the assay sensitivity was 1.0 pg/mL, and the manufacturer-reported intra- and inter-assay CVs were both <15%. The kit was supplied by Guangzhou Aoruida Biotechnology Co., Ltd., Guangzhou, China. Serum concentration of 8-OHdG was measured in pg/mL without creatinine normalization. In this enzyme-linked immunosorbent assay (ELISA), wells for standards, blanks, and testing samples were designed. The samples and standards were introduced into their designated wells, followed by the application of horseradish peroxidase (HRP)-conjugate reagent and subsequent incubation. This procedure was followed by washing the plate and inverting it against a clean paper towel to ensure the thorough removal of the wash buffer. Chromogen A and chromogen B were subsequently introduced and gently mixed, followed by an additional incubation at 37 °C for a duration of 15 min. Upon completion, a stop solution was added, resulting in a colorimetric change from blue to yellow. The optical density was measured at a wavelength of 450 nm, utilizing a microplate reader (CLARIOstar ^plus^).

### 2.9. Statistical Analysis

Conventional descriptive statistics were used to explain the characteristics of participants in the pre-burning and post-burning periods. The sample size of 152 participants was calculated using a paired Wilcoxon signed-rank test, assuming a standardized effect size of 0.23 and a two-sided significance level (α) of 0.05; the final sample size of 152 provided an estimated statistical power of 78.6% (β = 0.214), based on the Guenther approximation.

A comparison of 1-OHP and the oxidative stress biomarker between the pre- and post-burning periods was performed, and the *p*-value was calculated using the Wilcoxon signed-rank test because biomarker concentrations are typically not normally distributed. The *p*-values for age and body mass index were calculated by one-way ANOVA. The *p*-values for sex and smoking history were calculated using the Chi-square test. Correlations between within-person changes in urinary 1-OHP and oxidative stress biomarkers were calculated using Spearman’s rank correlation coefficients. Based on knowledge, we have categorized the biomarker comparison into three categories: low, moderate and high. However, for awareness and behavior, we have categorized the biomarker comparison into low and high. The *p*-values for the comparison of biomarkers related to knowledge, behavior and awareness were calculated using a paired *t*-test. Knowledge-based oxidative stress biomarkers were categorized into three subgroups, low, moderate and high knowledge, according to Bloom’s cut-off criteria, in which participants with scores equal to or more than 80% have high knowledge, participants with 60–79% have moderate knowledge, and participants with <60% have low knowledge [24]. The behavior- and awareness-based biomarker comparison knowledge scores were also dichotomized according to Bloom’s cut-off criteria. Participants with scores more than or equal to 80% were classified as high knowledge, while those with <80% were low knowledge. Questionnaires related to knowledge, awareness and behavior are shown in the Supplementary Materials, Sections S1.1–S1.3.

## 3. Results

### 3.1. Characteristics of Participants and Oxidative Stress Biomarkers

A total of 153 participants were recruited, of which 152 participated in both visits. These comprised healthy individuals (*n* = 88), participants with diabetes mellitus only (DM; *n* = 35), with hypertension only (HT; *n* = 15), and with combined DM and HT (*n* = 14). Supplementary Figure S1 shows a STROBE flow diagram of participant recruitment, inclusion and exclusion criteria.

Different participants groups have different means of age, which were (57.09 ± 8.03) years in healthy individuals, (61.49 ± 6.49) years in the DM group, (62.67 ± 11.06) years in the HT group, and (63.93 ± 5.48) years in the DM + HT group (overall: 59.28 ± 8.23 years; *p* = 0.001) as illustrated in (Table 1). Gender distribution also differed significantly among different groups of participants (*p* = 0.006), whereas smoking history (*p* = 0.445) and body mass index were comparable, with mean BMI values of 24.04 ± 3.64, 24.70 ± 4.13, 24.29 ± 3.06, and 25.01 ± 4.19 kg/m^2^ across the healthy, DM, HT, and DM + HT groups, respectively (overall: 24.31 ± 3.74 kg/m^2^), with *p* = 0.733, as shown in Table 1.

Biomarkers showed no significant differences among clinical groups during either the pre- or post-burning periods. Urinary 1-hydroxy pyrene values were 0.25 ± 0.66, 0.18 ± 0.25, 0.17 ± 0.12, and 0.13 ± 0.08 μmol/mol creatinine in healthy individuals and the DM, HT, and DM + HT groups, respectively (overall: 0.22 ± 0.51; *p* = 0.532), and after the post-burning period, 1-OHP values were 0.95 ± 1.39, 0.77 ± 1.06, 1.00 ± 1.34, and 0.63 ± 0.74 μmol/mol creatinine, respectively (overall: 0.88 ± 1.26; *p* = 0.585). Serum MDA did not show any significant difference during pre-burning (5.70 ± 3.77, 7.00 ± 3.91, 4.96 ± 2.72, and 7.03 ± 2.94 nmol/mL; overall: 6.10 ± 3.71; *p* = 0.063) or post-burning (6.07 ± 3.98, 5.36 ± 3.70, 6.04 ± 1.84, and 7.14 ± 3.30 nmol/mL; overall: 6.00 ± 3.72; *p* = 0.159), as shown in Table 1. Another serum biomarker is 8-OHdG, the values of which, among different participant groups during pre-burning, were 46.99 ± 9.52, 50.97 ± 7.72, 49.28 ± 6.80, and 64.19 ± 54.52 pg/mL (overall: 49.71 ± 18.69; *p* = 0.101) and, during post-burning, were 62.32 ± 12.32, 65.45 ± 9.05, 66.43 ± 11.85, and 74.77 ± 60.33 pg/mL (overall: 64.55 ± 21.07; *p* = 0.463). Overall, age and gender differed significantly among the clinical groups, whereas 1-OHP, MDA, and 8-OHdG levels were comparable despite slight numerical differences.

### 3.2. Comparison of Biomarkers (Urinary 1-OHP, Serum MDA and Serum 8-OHdG) Between Pre- and Post-Burning Periods Among Healthy, Diabetic, and Hypertension Groups

Urinary 1-OHP increased significantly in the post-burning season compared to the pre-burning season, increasing from (0.22 ± 0.51 to 0.88 ± 1.26 μmol/mol creatinine) (*p* < 0.001). This increasing behavior were seen in all participants, including healthy participants (0.25 ± 0.66 to 0.95 ± 1.39; *p* < 0.001), participants with diabetes mellitus only (0.18 ± 0.25 to 0.77 ± 1.06; *p* < 0.001), hypertension only (0.17 ± 0.12 to 1.00 ± 1.34; *p* = 0.006), and combined diabetes and hypertension (0.13 ± 0.76 to 0.63 ± 0.74; *p* = 0.003) as shown in Table 2.

The same pattern was seen for serum 8-OHdG in Table 2, which shows a significant increase in all participants from 49.71 ± 18.69 to 64.55 ± 21.07 pg/mL (*p* < 0.001). In healthy participants, 8-OHdG levels increased significantly from 46.99 ± 9.52 to 62.32 ± 12.32 (*p* < 0.001); those with diabetes mellitus only showed 50.97 ± 7.72 to 65.45 ± 9.05) (*p* < 0.001), with hypertension only showed 49.28 ± 6.80 to 66.43 ± 11.85 (*p* = 0.005), and with combined diabetes and hypertension showed 64.19 ± 54.32 to 74.77 ± 60.33 (*p* = 0.05). However, serum MDA did not show any significant change in the post-burning period (6.09 ± 3.71 to 6.00 ± 3.71 nmol/mL; *p* = 0.825). However, subgroup analyses similarly showed no significant change in healthy participants, the hypertension-only group, or the combined diabetes–hypertension group. Table 2 shows that MDA decreased significantly among participants with diabetes mellitus only (from 7.00 ± 3.91 to 5.36 ± 3.70 nmol/mL; *p* = 0.017). Collectively, these findings indicate that the burning period was accompanied by higher polycyclic aromatic hydrocarbon exposure and oxidative DNA damage, while lipid peroxidation remained comparatively unchanged.

Table 3 shows both creatinine-adjusted and -unadjusted urinary 1-OHP concentrations and urinary creatinine concentrations for the pre- and post-burning periods. Unadjusted urinary 1-OHP did not differ significantly between the two seasons (*p* = 0.161), whereas urinary creatinine concentrations were significantly lower during the post-burning period (*p* < 0.001).

### 3.3. Correlation of Urinary 1-OHP and Oxidative Stress Biomarkers

Table 4 illustrates that the change in urinary 1-OHP was not significantly correlated with the change in 8-OHdG (ρ = −0.050, BCa 95% CI: −0.210 to 0.107, n = 138, *p* = 0.557), whereas a weak negative correlation was observed between Δ1-OHP and ΔMDA (ρ = −0.204, BCa 95% CI: −0.352 to −0.054, n = 138, *p* = 0.016). The correlation between Δ8-OHdG and ΔMDA was not significant (ρ = 0.147, BCa 95% CI: −0.015 to 0.299, n = 138, *p* = 0.085).

**Figure 3:**
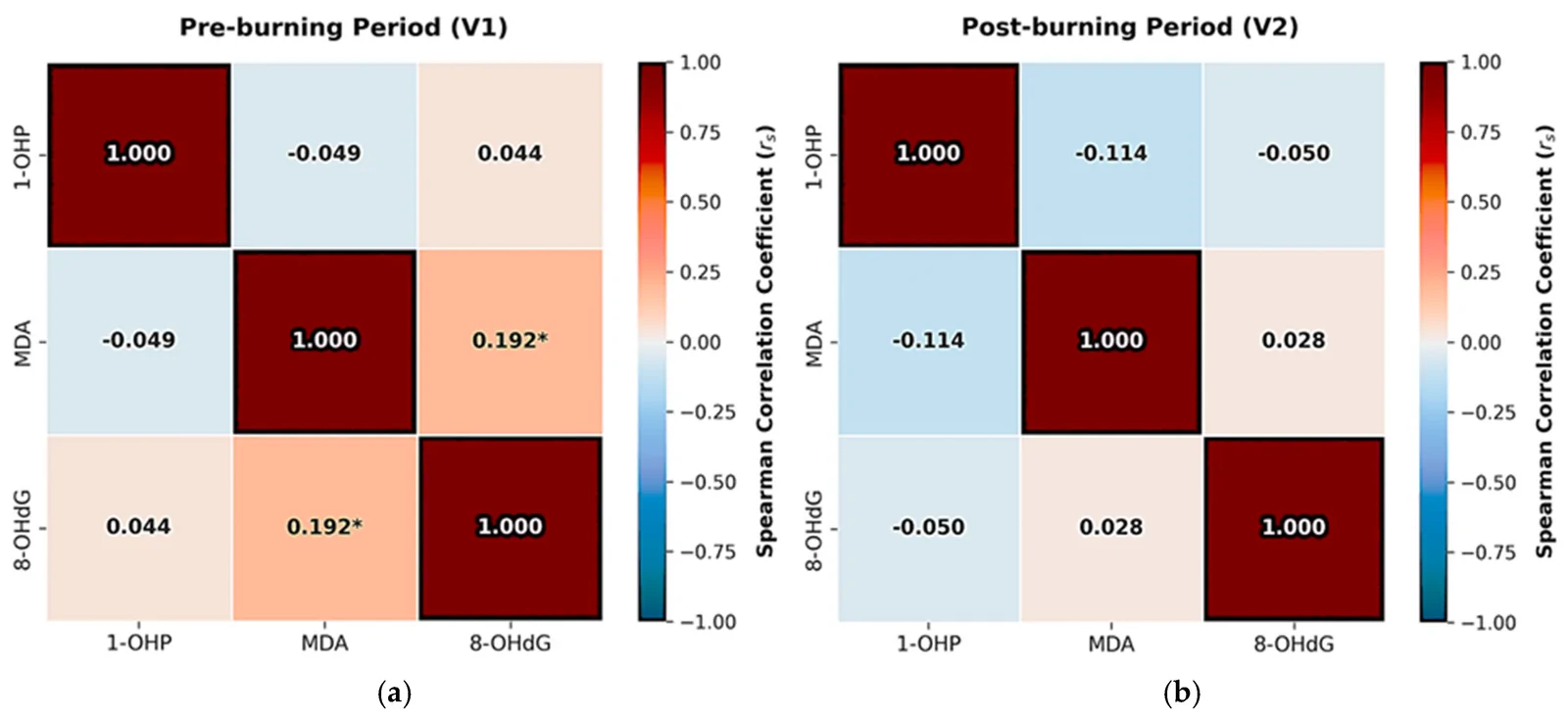
This figure is a Spearman correlation heat map. (**a**) Correlation of biomarkers in pre-burning season. (**b**) Correlation of biomarkers in post-burning season. (Abbreviations: 1-OHP: 1-hydroxypyrene; MDA: malondialdehyde; 8-OHdG: 8-hydroxy-2-deoxyguanosine).

Figure 3 shows a weak correlation between urinary 1-OHP and serum MDA and 8-OHdG. Most Spearman correlations were close to zero, which means these biomarkers have minimal association. Overall, no significant relationships were observed between PAH exposure (1-OHP) and the oxidative stress biomarkers, suggesting that these biomarkers varied independently in the study population.

**Figure 4:**
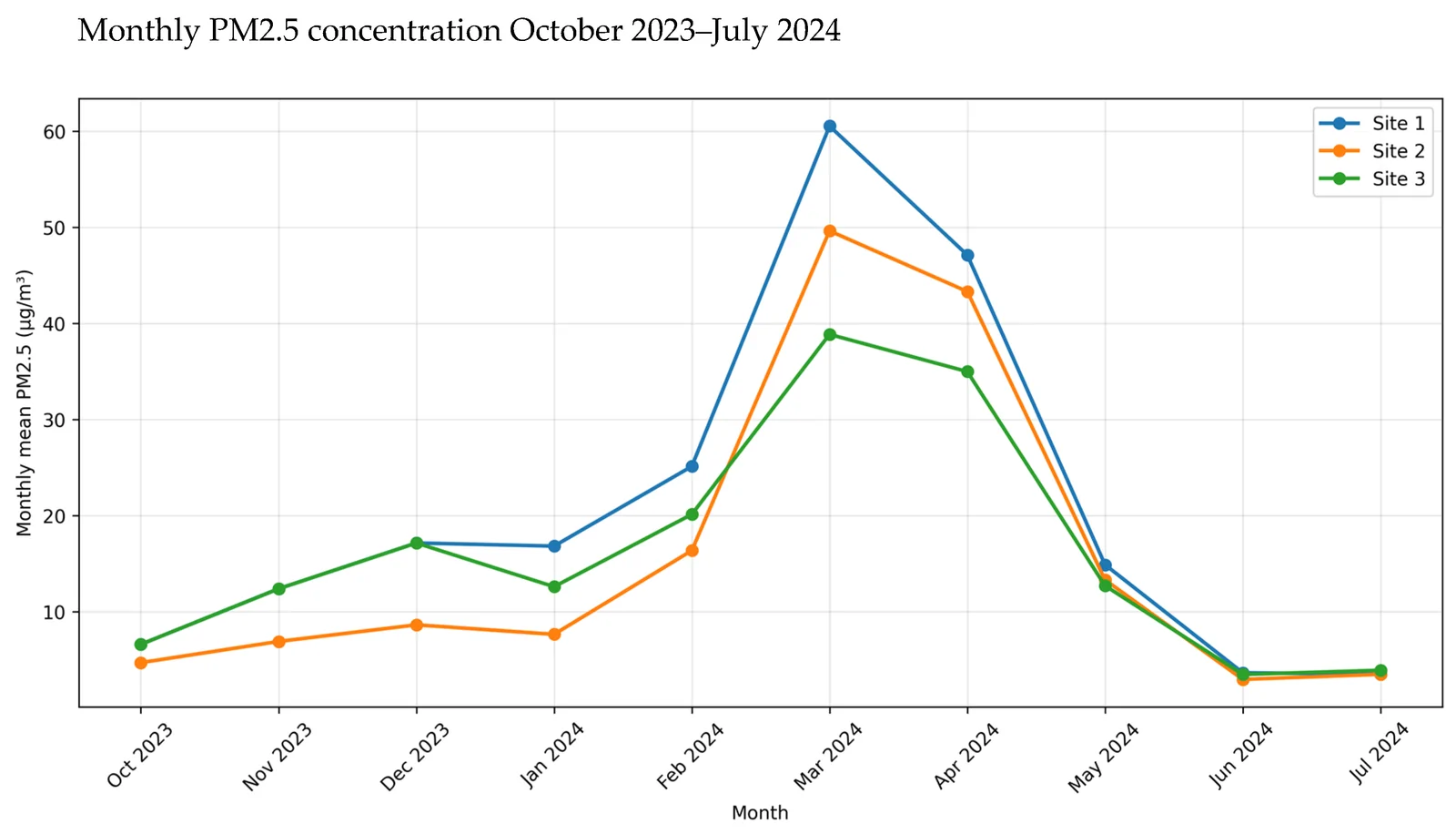
Average PM2.5 concentration (μg/m^3^) from October 2023–July 2024 across three study sites. This figure gives the monthly average PM2.5 concentration, with its peak in March 2024.

Figure 4 shows that the average PM2.5 concentration is low until January 2024, after which it started to increase, with a peak in March 2024. After March 2024, PM 2.5 started to decline. Supplementary Table S2 represents the average monthly PM2.5 concentration.

According to diabetes status, baseline characteristics are different. Table 5 shows that diabetic participants were greater in age compared to non-diabetic participants, 64.0 (58.0–66.0) vs. 58.0 (53.5–63.0) years (*p* = 0.001), and total cholesterol levels were also high in diabetics compared to non-diabetics: 193.0 (175.0–227.0) vs. 173.0 (156.5–199.0 mg/dL), (*p* = 0.002). Glucose levels in diabetics were also high compared to non-diabetics, 125.0 (104.5–141.0) vs. 101.0 (94.0–108.0) (*p* < 0.001), and systolic blood pressure was high (138.63 ± 16.15 vs. 131.27 ± 19.85 mmHg, *p* = 0.025). Exercise frequency between the diabetic and non-diabetic groups was significantly different (*p* = 0.004). However, Table 5 shows a comparison of BMI (24.79 ± 4.10 vs. 24.08 ± 3.55 kg/m^2^, *p* = 0.279), waist circumference (84.10 ± 10.94 vs. 81.88 ± 14.75 cm, *p* = 0.350), diastolic blood pressure (80.47 ± 13.51 vs. 79.50 ± 10.65 mmHg, *p* = 0.634), smoking status (*p* = 0.550), and alcohol consumption (*p* = 0.882) between diabetic and non-diabetic participants.

Linear mixed-effects models showed significant seasonal effects for 1-OHP and 8-OHdG (both *p* < 0.001), indicating significant differences in biomarker levels between the pre-burning and post-burning seasons after adjustment for age, sex, BMI, and smoking history, as shown in Table 6. In contrast, MDA showed no significant seasonal effect (*p* = 0.969). No significant differences were observed among disease groups for 1-OHP (*p* = 0.658), 8-OHdG (*p* = 0.171), or MDA (*p* = 0.482). Furthermore, the group × season interaction was not significant for 1-OHP (*p* = 0.880), 8-OHdG (*p* = 0.369), or MDA (*p* = 0.097), indicating that the magnitude of seasonal changes did not differ significantly across disease groups, as illustrated in Table 6.

### 3.4. Characteristics of Biomarkers (Urinary 1-OHP, Serum MDA and Serum 8-OHdG) in Different Symptoms of Participants

Supplementary Table S1 presents an exploratory comparison of urinary 1-OHP, serum 8-OHdG, and MDA between the pre- and post-burning periods according to self-reported symptoms. MDA did not differ significantly between the two periods for any symptom category after FDR adjustment. The changes in MDA were small across the symptom strata, with adjusted *p*-values of 1.000 for all comparisons.

In contrast, 8-OHdG increased significantly during the high-burning period among participants reporting eye irritation (52.14 ± 29.62 to 67.23 ± 32.03 pg/mL), skin irritation (49.94 ± 24.21 to 65.11 ± 26.80 pg/mL), respiratory irritation (54.53 ± 36.34 to 67.83 ± 40.36 pg/mL), shortness of breath (51.46 ± 26.81 to 63.47 ± 28.67 pg/mL), and feeling dizzy (53.60 ± 32.58 to 65.69 ± 35.14 pg/mL), with all FDR-adjusted *p*-values < 0.001. Among participants reporting loss of consciousness, 8-OHdG also increased from 51.51 ± 10.94 to 65.43 ± 9.46 pg/mL (FDR-adjusted *p* = 0.012). A significant increase was also observed among participants reporting pneumonia, from 47.29 ± 10.55 to 61.94 ± 11.99 pg/mL (FDR-adjusted *p* < 0.001). In contrast, the change among participants reporting fainting was not significant (54.01 ± 10.61 to 63.07 ± 8.02 pg/mL; FDR-adjusted *p* = 0.169), as shown in Supplementary Table S1.

Urinary 1-OHP showed a similar pattern, with significantly higher concentrations during the high-burning period among participants reporting eye irritation, skin irritation, respiratory irritation, shortness of breath, feeling dizzy, and pneumonia (all FDR-adjusted *p*-values < 0.001). Among participants reporting loss of consciousness, 1-OHP increased from 0.16 ± 0.14 to 0.77 ± 0.46 μmol/mol creatinine (FDR-adjusted *p* = 0.012). No significant change was observed among participants reporting fainting (0.17 ± 0.14 to 0.76 ± 0.49 μmol/mol creatinine; FDR-adjusted *p* = 0.091).

### 3.5. Comparison of Oxidative Stress Biomarkers Between Pre- and Post-Burning Periods According to Knowledge-Related Responses

Supplementary Table S3 shows a comparison of biomarkers in pre- and post-burning according to knowledge. Across all knowledge groups, 8-OHdG levels increased significantly from the pre-burning to post-burning period, with *p* < 0.001 for high-, moderate-, and low-knowledge groups. Similarly, 1-OHP levels increased significantly in the high- (*p* < 0.001), moderate- (*p* = 0.001), and low- (*p* = 0.016) knowledge groups. In contrast, MDA levels did not show significant changes in the high- (*p* = 0.794), moderate- (*p* = 0.189), or low- (*p* = 0.674) knowledge groups.

### 3.6. Comparison of Oxidative Stress Biomarkers Between Pre- and Post-Burning Periods According to Awareness-Related Responses

We evaluated changes in oxidative stress biomarkers between the pre- and post-burning periods across low and high knowledge groups. MDA levels showed no statistically significant change after burning in either the low-knowledge group (*p* = 0.265) or the high-knowledge group (*p* = 0.385). In contrast, 8-OHdG levels increased significantly during the post-burning period in both the low-knowledge (*p* < 0.001) and high-knowledge (*p* < 0.001) groups. Similarly, 1-OHP levels elevated significantly post-burning across low-knowledge (*p* = 0.014) and high-knowledge (*p* < 0.001) participants. Overall, 8-OHdG and 1-OHP demonstrated significant post-burning increases regardless of awareness score, whereas MDA changes remained non-significant, as shown in Supplementary Table S4.

### 3.7. The Characteristics of Oxidative Stress Biomarkers in Pre- and Post-Burning Periods According to Behavior-Related Responses

Supplementary Table S5 shows that 8-OHdG levels rose significantly post-burning in both the low (*p* < 0.001) and high (*p* < 0.001) groups. Similarly, 1-OHP levels increased significantly following the burning period for both the low (*p* = 0.001) and high (*p* < 0.001) behavior categories. Conversely, MDA levels showed no significant change post-burning in either the low group (*p* = 0.667) or the high group (*p* = 0.812).

## 4. Discussion

While this investigation concentrated on Chiang Mai, a location distinguished by its unique geographic and agricultural attributes (e.g., mountainous topography and seasonal combustion), the results may not be readily extrapolated to other populations or nations characterized by divergent climatic conditions and sources of pollution. During high PM2.5 periods, haze can be seen in Northern Thailand, which is commonly due to extensive biomass burning and the burning of agricultural residue during the dry season [25]. Polycyclic aromatic hydrocarbons represent hazardous contaminants, as exposure to these detrimental agents elevates oxidative stress levels, thereby instigating and exacerbating numerous health conditions, including diabetes and hypertension.

A study showed that exposure to polycyclic aromatic hydrocarbons (PAHs) expedited the production of reactive oxygen species (ROS), which can damage DNA by formation of DNA adducts [26]. A multitude of research investigations have demonstrated that 8-OHdG serves not only as a biomarker for oxidative stress, but also represents a potential risk factor for malignancies, coronary artery disease, diabetes mellitus, and atherosclerotic conditions, in addition to functioning as a prognostic indicator for the progression and outcome of certain diseases [27]. The Wilcoxon signed-rank test showed that urinary 1-OHP and serum 8-OHdG levels were significantly higher during the post-burning period than during the pre-burning period with (*p* < 0.001). Previous studies showed that air pollution causes an increase in urinary 1-OHP and 8-OHdG, which are oxidative DNA damage biomarkers [28].

In contrast to 8-OHdG, MDA remained unchanged. The contrast is because 8-OHdG reflects early oxidative DNA damage following short-term exposure, whereas lipid peroxidation does not. The lack of a significant increase in MDA may be due to the body’s natural antioxidant defense system, including superoxide dismutase, catalase, and glutathione peroxidase, which helps protect cells from oxidative damage. In the early phase or short-term PM2.5 exposure, these antioxidant responses may limit lipid peroxidation before more extensive oxidative damage occurs [29]. Circulating MDA was influenced by diet, lipid metabolism, and antioxidant status, and its measurements using the TBARS assay are subject to limited specificity and greater analytical variability. TBARSs lack specificity for MDA and is susceptible to interference from other thiobarbituric acid-reactive compounds; it may be less sensitive for detecting subtle changes in lipid peroxidation following short-term PM2.5 or PAH exposure. Lipid peroxidation generally reflects more extensive or sustained oxidative injury, whereas DNA damage occurs earlier during exposure to combustion-related air pollution.

In this study, we also found that the higher level of 8-OHdG in diabetes and hypertension, as compared to healthy control groups, was similar to that of the study by Nemtsova et al., 2022 [30]. However, in contrast to the study by Premkumar et al., 2023 [31], in which their study showed a significant increase in lipid peroxidation, our study displayed a significant decrease in the lipid peroxidation biomarker MDA. However, our findings of unchanged MDA are consistent with a study by Lee et al. (2024), in which MDA was not associated with either personal or ambient PM2.5 exposure [32]. MDA in diabetic groups has decreased significantly (*p* = 0.017). The significant decrease in MDA observed in patients with diabetes in the post-burning season is unexpected; one possible reason could be the antioxidant capacity, which could limit lipid peroxidation. The antioxidant capacity is still uncertain because we did not measure antioxidants like catalase, superoxide dismutase, etc., in participants. The other possible reason could be that we neither have medication data for diabetic patients nor do we have any other data on diet and other supplements rich in antioxidants, which could limit peroxidation despite increased environmental oxidative stress. One study revealed that curcumin and vitamin D significantly decreased malondialdehyde (MDA) levels [33]. A third possible explanation could be the limited specificity of TBARSs for measuring MDA.

In the individuals diagnosed with diabetes, levels of 1-OHP exhibited a notable elevation in comparison to measurements taken prior to exposure to combustion-related air pollution and after such exposure. In the present investigation, we found that oxidative DNA damage biomarkers such as 8-OHdG rose significantly across the burning season in every clinical group. A relevant study revealed that individuals diagnosed with diabetes, who experienced brief episodes of pulmonary arterial hypertension, were significantly affected by oxidative stress [34]. We also found that urinary 1-OHP concentration and serum 8-OHdG were significantly increased in the post-burning period compared to the pre- burning period in diabetic groups.

The urinary 1-OHP and oxidative stress biomarkers had minimal individual association. As we did not have patients’ antioxidant status, medication data or diet data, this weak correlation is tentative. 1-OHP is an exposure to PAH, while oxidative stress depends on lifestyle, diet, antioxidant capacity and medication data. Individual differences in lifestyle, cooking practices, exposure to traffic and dietary habits may contribute to weakening the association between PAH exposure and oxidative stress [9].

All the healthy, diabetic, and hypertension patients and those with both diabetes and hypertension showing symptoms like eye irritation, skin irritation, respiratory irritation, shortness of breath, dizziness, fainting and pneumonia had higher 8-OHdG levels in the post-burning period compared to the pre-burning period. In contrast to 8-OHdG, MDA showed irregular patterns in all kinds of symptoms of all types of subgroups.

Diabetic dermopathy is a skin condition commonly observed in individuals with diabetes. One investigation indicated that individuals diagnosed with diabetes who experience dermatological irritations were predisposed to complications related to infections such as fungal and bacterial pathogens [35]. A separate investigation revealed that exposure to atmospheric pollutants intensified dermal irritation among individuals with diabetes because of their heightened sensitivity to environmental contaminants [36]. The other study reported that individuals diagnosed with diabetes frequently encounter severe complications, including cardiovascular disorders, obesity, and respiratory ailments. Those with diabetes who exhibited symptoms of dyspnea demonstrated an elevated risk when subjected to polycyclic aromatic hydrocarbons [37].

The findings have important implications for public health and air quality management in Northern Thailand, where biomass burning contributes to major PM2.5 and PAH exposure. A study from upper Northern Thailand revealed that the implementation of burning restrictions has reduced particulate matter pollution and decreased hospital visits for respiratory diseases [38]. Alongside regulation of policy on open burning restrictions, improved household cooking technologies according to the policy may help reduce indoor air pollution, particularly in communities relying on biomass fuels [39].

The first limitation of this study was the generalizability of the population, as participants were recruited in specific areas of Chiang Mai province, which could have different environmental conditions and sources of air pollution. This study did not include a clearly defined unexposed control group, which limits our ability to distinguish the effects of biomass-burning-related PM2.5 exposure from background oxidative stress and other seasonal or environmental influences. Although the pre- and post-burning-season design allowed for the assessment of within-participant changes, the observed changes in biomarkers cannot be attributed exclusively to biomass-burning exposure. Other factors, including underlying chronic diseases, medication use, smoking, lifestyle factors, and co-exposure to other pollutants, may have also contributed to biomarker variability. Medications used to manage these conditions may influence oxidative stress, inflammation, and metabolic pathways and consequently affect 8-OHdG, MDA, and other biomarker levels. Therefore, some differences in biomarker levels may be related to medication use rather than being caused only by PM2.5 exposure. In this study, only two biomarkers, MDA and 8-OHdG, were evaluated; other biomarkers of oxidative stress, inflammation and antioxidant defense mechanisms were not evaluated, but they may provide a more comprehensive understanding of air pollution exposure. 8-OHdG was measured using a competitive ELISA, which may have lower analytical specificity and cross-reactivity with other oxidized guanine products compared with LC-MS/MS. Therefore, the 8-OHdG findings should be interpreted cautiously, and confirmation using LC-MS/MS is warranted in future studies.

Another limitation of this study is the use of a non-validated knowledge, awareness and behavior scale. Another study demonstrated adequate reliability for assessing environmental health knowledge among healthcare professionals [40]. The KAB analyses involved prespecified pre- and post-burning-season comparisons, while Spearman analyses were exploratory biomarker correlations; therefore, FDR adjustment was not applied. Future studies should consider employing the validated knowledge scale to improve measurement reliability and comparability. Future studies should include further oxidative stress biomarkers, inflammatory biomarkers and antioxidant defense mechanisms. Further research is also needed to identify the specific PAH compound that causes oxidative stress and evaluate the effectiveness of preventive interventions in reducing air pollution exposure.

## 5. Conclusions

This study revealed that seasonal biomass burning in Chiang Mai caused exposure to polycyclic aromatic hydrocarbons, as proven by elevated levels of urinary 1-hydroxypyrene (1-OHP) in the post-burning season. Seasonal burning activities in Chiang Mai exhibit a correlation with elevated polycyclic aromatic hydrocarbon (PAH) exposure, as well as preliminary indications of oxidative stress, with 8-hydroxy-2′-deoxyguanosine (8-OHdG) serving as a sensitive biomarker for DNA damage. Patients with diabetes and hypertension showed greater baseline levels of oxidative stress than healthy individuals.

In contrast to oxidative DNA damage, MDA did not differ between the pre- and post-burning periods, which means that lipid peroxidation is less sensitive to short-term air pollution exposure compared to oxidative DNA damage. Lipid peroxidation typically serves as an indicator of more protracted or severe oxidative damage, whereas DNA damage manifests at an earlier stage during exposure to air pollution associated with combustion. Circulating MDA was influenced by some factors such as dietary intake, lipid metabolic processes, and antioxidant status, and measurements of MDA using the TBARS assay are subject to limited specificity.

In the post-burning period, vulnerable populations should be prioritized through targeted notifications. In outdoor environments, N95 respirators should be used, and indoor air filtration systems should be implemented. In the management of diabetes and hypertension, the 1-hydroxypyrene and oxidative stress biomarkers, particularly 8-OHdG, should be assessed. At the policy level, governmental bodies should prohibit biomass burning rather than undertake initiatives to manage these residues. Future research endeavors should establish connections between these biomarkers and clinical outcomes, while enhancing exposure assessments through the speciation of PAHs and personal monitoring techniques.

## Figures and Tables

**Table 1:** Characteristics of participants in all clinical groups.

Variables		Healthy (88)	DM Only (35)	HT Only (15)	DM + HT (14)	*p*-Value	Total (N = 152)
Age (years, mean ± SD )		57.09 ± 8.03	61.49 ± 6.49	62.67 ± 11.06	63.93 ± 5.48	*p* = 0.001	59.28 ± 8.23
Sex (n, %)						*p* = 0.006	
Male		69 (78.4%)	31 (88.6%)	9 (60%)	8 (57.1%)		117 (77.1%)
Female		19 (21.6%)	4 (11.4%)	6 (40%)	6 (42.9%)		35 (22.9%)
Smoking history (n, %)						*p* = 0.445	
Yes		8 (9.1%)	2 (5.7%)	3 (20%)	1 (7.1%)		14 (9.2%)
No		80 (90.9%)	33 (94.3%)	12 (80%)	13 (92.9%)		138 (90.8%)
Body mass index (mean ± SD)		24.04 ± 3.64	24.70 ± 4.13	24.29 ± 3.06	25.01 ± 4.19	*p* = 0.733	24.31 ± 3.74
Urinary 1-OHP (μmol/mol Cre)	Pre- burning	0.25 ± 0.66	0.18 ± 0.25	0.17 ± 0.12	0.13 ± 0.08	*p* = 0.532	0.22 ± 0.51
	Post-burning	0.95 ± 1.39	0.77 ± 1.06	1.00 ± 1.34	0.63 ± 0.74	*p* = 0.585	0.88 ± 1.26
Serum MDA (nmol/mL mean ± SD)	Pre-burning	5.70 ± 3.77	7.00 ± 3.91	4.96 ± 2.72	7.03 ± 2.94	*p* = 0.063	6.10 ± 3.71
	Post-burning	6.07 ± 3.98	5.36 ± 3.70	6.04 ± 1.84	7.14 ± 3.30	*p* = 0.159	6.00 ± 3.72
Serum 8-OHdG (pg/mL mean ± SD)	Pre-burning	46.99 ± 9.52	50.97 ± 7.72	49.28 ± 6.80	64.19 ± 54.52	*p* = 0.101	49.71 ± 18.69
	Post-burning	62.32 ± 12.32	65.45 ± 9.05	66.43 ± 11.85	74.77 ± 60.33	*p* = 0.463	64.55 ± 21.07

Values are presented as mean ± SD or n (%). *p*-values for age and body mass index were calculated by one-way ANOVA. *p*-values for sex and smoking history were calculated using the chi-square test. *p*-values for urinary 1-OHP, serum MDA, and serum 8-OHdG were calculated using Kruskal–Wallis test. In Table 1, these *p*-values compare healthy, DM-only, HT-only and DM + HT groups within the same season. (Abbreviations: 1-OHP: 1-hydroxypyrene; MDA: malondialdehyde; 8-OHdG: 8-hydroxy-2-deoxyguanosine).

**Table 2:** Comparison of oxidative stress biomarkers in different clinical groups.

Group	N	1-OHP (μmol/mol Creatinine)		*p*-Value	MDA (nmol/mL)		*p*-Value	8-OHdG (pg/mL)		*p*-Value
		Pre-Burning Mean ± SD	Post-Burning Mean ± SD		Pre-Burning Mean ± SD	Post-Burning Mean ± SD		Pre-Burning Mean ± SD	Post-Burning Mean ± SD	
Healthy	88	0.25 ± 0.66	0.95 ± 1.39	<0.001	5.70 ± 3.77	6.07 ± 3.98	0.43	46.99 ± 9.52	62.32 ± 12.32	<0.001
DM only	35	0.18 ± 0.25	0.77 ± 1.06	<0.001	7.00 ± 3.91	5.36 ± 3.70	0.017	50.97 ± 7.72	65.45 ± 9.05	<0.001
HT only	15	0.17 ± 0.12	1.00 ± 1.34	0.006	4.96 ± 2.72	6.04 ± 1.84	0.53	49.28 ± 6.80	66.43 ± 11.85	0.005
DM + HT	14	0.13 ± 0.08	0.63 ± 0.74	0.003	7.03 ± 2.94	7.14 ± 3.30	0.433	64.19 ± 54.52	74.77 ± 60.33	0.05
All participants	152	0.22 ± 0.51	0.88 ± 1.26	<0.001	6.09 ± 3.71	6.00 ± 3.71	0.825	49.71 ± 18.69	64.55 ± 21.07	<0.001

Values are presented as mean ± SD. *p*-values were calculated using Wilcoxon signed-rank test. Abbreviations (1-OHP: 1-hydroxypyrene; MDA: malondialdehyde; 8-OHdG: 8-hydroxy-2-deoxyguanosine). Comparison of biomarkers in both seasons.

**Table 3:** Comparison of creatinine-adjusted and -unadjusted urinary 1-OHP and urinary creatinine concentrations between the pre- and post-burning seasons.

Variable	Pre-Burning	Post-Burning	*p*-Value
Creatinine-adjusted urinary 1-OHP (μmol/mol Cre.)	0.22 ± 0.51	0.88 ± 1.26	<0.001
Unadjusted urinary 1-OHP (ng/mL)	0.0284 (0.0129–0.0760)	0.0312 (0.0146–0.0719)	0.161
Urinary creatinine (mg/dL)	69.82 ± 39.51	24.92 ± 29.06	<0.001

Values are presented as mean ± SD or median (IQR), as appropriate. Differences between the pre- and post-burning periods were assessed using the Wilcoxon signed-rank test. (Abbreviations: 1-OHP: 1-hydroxypyrene).

**Table 4:** Correlations between within-person changes in urinary 1-OHP and oxidative stress biomarkers.

Variables	Spearman’s ρ	BCa 95% CI	*p*-Value
Δ1-OHP vs. Δ8-OHdG	−0.050	−0.210 to 0.107	0.557
Δ1-OHP vs. ΔMDA	−0.204	−0.352 to −0.054	0.016
Δ8-OHdG vs. ΔMDA	0.147	−0.015 to 0.299	0.085

Values represent Spearman’s rank correlation coefficients. Δ represents the within-person change from the pre-burning period to the post-burning period (post-burning minus pre-burning). The 95% confidence intervals were estimated using bias-corrected and accelerated (BCa) bootstrap intervals based on 5000 bootstrap samples. (Abbreviations: 1-OHP: 1-hydroxypyrene; MDA: malondialdehyde; 8-OHdG: 8-hydroxy-2-deoxyguanosine).

**Table 5:** Baseline characteristics of participants according to diabetes status.

Characteristics	Non-DM (n = 103)	DM (n = 49)	*p*-Value
Age, years	58.0 (53.5–63.0)	64.0 (58.0–66.0)	0.001
BMI, kg/m^2^	24.08 ± 3.55	24.79 ± 4.10	0.279
Waist circumference, cm	81.88 ± 14.75	84.10 ± 10.94	0.35
Total cholesterol, mg/dL	173.0 (156.5–199.0)	193.0 (175.0–227.0)	0.002
Glucose, mg/dL	101.0 (94.0–108.0)	125.0 (104.5–141.0)	<0.001
Systolic BP, mmHg	131.27 ± 19.85	138.63 ± 16.15	0.025
Diastolic BP, mmHg	79.50 ± 10.65	80.47 ± 13.51	0.634
Smoking status			0.55
└ Non-smoker	92 (89.3%)	46 (93.9%)	
└ Ever smoker	11 (10.7%)	3 (6.1%)	
Alcohol drinking			0.882
└ No	66 (64.1%)	32 (65.3%)	
└ Yes	37 (35.9%)	17 (34.7%)	
Exercise frequency			0.004
└ No exercise	37 (36.3%)	5 (10.2%)	
└ Irregular exercise	50 (49.0%)	33 (67.3%)	
└ Regular exercise	15 (14.7%)	11 (22.4%)	

Data are presented as mean ± SD, median (interquartile range, IQR), and number %, as appropriate. Age, total cholesterol, glucose, systolic blood pressure and exercise frequency were significantly different in diabetics and non-diabetics.

**Table 6:** Linear mixed model for calculation of *p*-value of oxidative stress biomarkers.

Outcome	Season Effect *p*-Value	Disease Group *p*-Value	Group × Season *p*-Value
1-OHP	<0.001	0.658	0.880
8-OHdG	<0.001	0.171	0.369
MDA	0.969	0.482	0.097

*p*-values were derived from linear mixed-effects models with participant-specific random intercepts, adjusting for age, sex, BMI, and smoking history. Season, disease group, and season × disease group interaction were included as fixed effects. (Abbreviations: 1-OHP: 1-hydroxypyrene; MDA: Malondialdehyde; 8-OHdG: 8-Hydroxy-2-deoxyguanosine).

## Data Availability

The data that supports the findings of this study can be obtained from the corresponding author upon reasonable request. Accordingly, we have prepared a de-identified dataset containing the biomarker measurements used in the analyses, including 1-OHP, MDA, and 8-OHdG, together with the relevant non-identifiable study variables.

## Supplementary Materials

The following supporting information can be downloaded at https://www.mdpi.com/article/10.3390/toxics14090771/s1: Supplementary Table S1: Exploratory comparison of urinary 1-OHP and oxidative stress biomarkers between low- and high-burning periods according to self-reported symptoms in all participant groups (with FDR-adjusted *p*-values). Supplementary Table S2: Supplementary Table S2 shows average PM2.5 (μg/m^3^) across three study sites from October 2023 to July 2024. Supplementary Materials Section S1.1: Knowledge questions. Supplementary Materials Section S1.2: Awareness questions. Supplementary Materials Section S1.3: Behavior questions. Supplementary Table S3: Association between knowledge level and oxidative stress biomarkers during pre- and post-burning periods. Supplementary Table S4: Association between awareness level and oxidative stress biomarkers during pre- and post-burning periods. Supplementary Table S5: Association between behavior level and oxidative stress biomarkers during pre- and post- burning periods. Supplementary Figure S1: Supplementary Figure S1 shows a STROBE flow diagram of participants recruitment, inclusion, and exclusion criteria.

## Abbreviations

The following abbreviations were used in this manuscript:

PAHs	Polycyclic Aromatic Hydrocarbons
PM2.5	Particulate Matter with Diameter Less Than 2.5 µm
1-OHP	1-Hydroxypyrene
MDA	Malondialdehyde
8-OHdG	8-Hydroxy-2-Deoxyguanosine
NTAQHI	Northern Thailand Air Quality Health Index
ELISA	Enzyme-Linked Immunosorbent Assay
HPLC	High-Performance Liquid Chromatography
DM	Diabetes Mellitus
HT	Hypertension
POCT	Point-of-Care Testing
