# Supplementary material for: The Impact of Polycyclic Aromatic Hydrocarbons Exposure from Seasonal Biomass Burning on Oxidative Stress and Genotoxicity Biomarkers in Patients with Diabetes and Hypertension in Chiang Mai, Thailand
Source: Toxics. 2026 Aug 29;14(9):771. doi: 10.3390/toxics14090771 (PMC13611676; doi:10.3390/toxics14090771)

Supplementary Table S1. Exploratory comparison of urinary 1-OHP and oxidative stress biomarkers between low- and high-burning periods according to self-reported symptoms in all participant groups (with FDR-adjusted p-values).

Symptom	N	MDA pre-burning (nmol/mL)	MDA post-burning (nmol/mL)	MDA Raw-p-value	MDA FDR-p-value	8-OHdG pre-burning (pg/mL)	8-OHdG post-burning (pg/mL)	8-OHdG Raw p- value	8-OHdG FDR p-value	1-OHP pre-burning (µmol/mol creatinine)	1-OHP post-burning (µmol/mol creatinine)	1-OHP Raw p-value	1-OHP FDR p-value
Eye irritation	105	6.29±3.80	5.94±3.84	0.441	1.000	52.14±29.62	67.23±32.03	<0.001	<0.001	0.15±0.15	0.79±1.18	<0.001	<0.001
Skin irritation	80	5.69±3.35	5.80±3.40	0.895	1.000	49.94±24.21	65.11±26.80	<0.001	<0.001	0.16±0.20	0.83±1.32	<0.001	<0.001
Respiratory irritation	67	5.89±3.40	5.76±3.37	0.656	1.000	54.53±36.34	67.83±40.36	<0.001	<0.001	0.15±0.15	0.82±1.27	<0.001	<0.001
Shortness of breath	68	6.27±3.67	6.25±3.67	0.741	1.000	51.46±26.81	63.47±28.67	<0.001	<0.001	0.16±0.21	0.81±1.36	<0.001	<0.001
Feeling dizzy	43	5.63±2.94	5.64±3.25	0.953	1.000	53.60±32.58	65.69±35.14	<0.001	<0.001	0.15±0.13	0.95±1.54	<0.001	<0.001
Fainting	11	5.07±2.76	6.67±2.32	0.333	1.000	54.01±10.61	63.07±8.02	0.169	0.169	0.17±0.14	0.76±0.49	0.070	0.091
Losing consciousness	16	5.17±2.40	5.90±2.34	0.532	1.000	51.51±10.94	65.43±9.46	0.009	0.012	0.16±0.14	0.77±0.46	0.010	0.012
Having pneumonia	60	5.72±3.30	5.85±3.30	0.813	1.000	47.29±10.55	61.94±11.99	<0.001	<0.001	0.17±0.22	0.84±1.32	<0.001	<0.001

Values are presented as mean ± SD. Differences between pre- and post-burning seasons within each symptom subgroup were analyzed using the Wilcoxon signed-rank test. False discovery rate (FDR) correction was performed using the Benjamini–Hochberg procedure. An FDR-adjusted p-value < 0.05 was considered statistically significant.

Supplementary Table S2. Supplementary Table 2 shows average PM2.5 (μg/m³) across three study sites from Oct. 2023 to July 2024.

	**Oct 2023**	**Nov-2023**	**Dec-2023**	**Jan-2024**	**Feb-2024**	**March-2024**	**April-2024**	**May-2024**	**June-2024**	**July-2024**
**Site 1**	6.61	12.40	17.16	16.84	25.14	60.55	47.13	14.90	3.66	3.50
**Site 2**	4.71	6.92	8.65	7.66	16.38	49.65	43.33	13.32	2.97	3.50
**Site 3**	6.61	12.40	17.16	12.61	20.14	38.87	35.00	12.71	3.48	3.92

S1. Supplementary Materials: Knowledge, Awareness, and Behavior (KAB) Questionnaire Used in the Study

The questionnaire for knowledge, awareness and behavior is as follows.

S1.1. Knowledge questions

1: The cause of the smog problem in Chiang Mai is all kinds of burning; **2:** Open burning is one of the causes of dirty air because there are contaminants in the air such as smoke, dust; **3:** Chiang Mai City is located in a lowland basin so the air is not well-ventilated, causing dust to cover the city; **4:** During winter, the air is still and low, causing more pollution than at

other times; **5:** Dust from construction or factories must be removed before being released into the atmosphere; **6:** Fine particles from various types of burning can damage the respiratory system of people; **7:** People in areas with polluted air are more likely to suffer from lung diseases than people in other areas; **8:** Air pollution causes soil and water quality to deteriorate; **9:** Smog causes traffic accidents; **10:** Smog causes poor visibility in flights, preventing planes from taking off or landing; **11:** Chiang Mai loses a lot of economic income from air pollution; **12:** People lose a lot of health care expenses from air pollution; **13:** Local officials have the power to order a ban on open burning. Those who do not comply with the order may be punished.

S1.2. Awareness questions

1: Burning pollutes the air in the area where it is burned and spreads the pollution to other areas; **2:** All types of burning make the air in Mueang Chiang Mai hotter; **3:** Reducing the amount of garbage in your home reduces air pollution; **4:** Repairing and reusing damaged items reduces air pollution; **5:** Sorting garbage before throwing it away can solve the problem of air pollution; **6:** Everyone should avoid burning; **7:** Everyone should help plant trees; **8:** Everyone should help monitor garbage burning in the community.

S1.3. Behavior questions

1: Having an accident caused by smog on the roadside; **2:** Avoid outdoor exercise or stay in open areas; **3:** Avoid burning of any kind; **4:** Reduce the amount of household waste; **5:** Use water to wash around the house to reduce the amount of dust; **6:** Close the doors and windows of the house to prevent dust; **7:** Help plant trees; **8:** If driving a vehicle, turn off the engine when parked; **9:** Have an annual health check to monitor health.

Supplementary Table S3. Association between knowledge level and oxidative stress biomarkers during pre- and post-burning periods.

Questions (Knowledge) score	N	MDA (nmol/mL) Pre-burning	MDA (nmol/mL) Post-burning	p-value	8-OHdG (pg/mL) Pre-burning	8-OHdG (pg/mL) Post-burning	p-value	1-OHP (µmol/mol creatinine) Pre-burning	1-OHP (µmol/mol creatinine) Post-burning	p-value
High knowledge (11-13)	80	6.31 ± 4.00	6.43 ± 3.90	0.794	48.94 ± 9.85	61.19 ± 12.55	<0.001	0.28 ± 0.67	0.94 ± 1.34	<0.001
Moderate knowledge (8-10)	37	5.91 ± 3.68	4.97 ± 3.05	0.189	52.53 ± 33.51	69.84 ± 34.77	<0.001	0.14 ± 0.11	1.01 ± 1.38	0.001
Low knowledge (<8)	20	5.89 ± 2.93	6.26 ± 3.79	0.674	47.78 ± 8.74	68.23 ± 8.37	<0.001	0.15 ± 0.22	0.50 ± 0.57	0.016

Data are presented as mean ±SD. *p*-values were calculated using a paired t-test. *p*-values <0.05 indicate statistically significant differences between pre- and post-burning measurements.

Supplementary Table S4. Association between awareness level and oxidative stress biomarkers during pre- and post-burning periods.

Questions (Awareness) score	N	MDA (nmol/mL) Pre-burning	MDA (nmol/mL) Post-burning	p-value	8-OHdG (pg/mL) Pre-burning	8-OHdG (pg/mL) Post- burning	p-value	1-OHP (µmol/mol creatinine) Pre-burning	1-OHP (µmol/mol creatinine) Post-burning	p-value
Low knowledge (<7)	24	5.06 ± 2.82	5.93 ± 2.93	0.265	55.22 ± 41.72	77.44 ± 42.96	<0.001	0.10 ± 0.09	0.38 ± 0.52	0.014
High knowledge (7-8)	115	6.38 ± 3.90	6.02 ± 3.88	0.385	48.77 ± 9.96	61.91 ± 11.33	<0.001	0.25 ± 0.57	0.99 ± 1.35	<0.001

Data are presented as mean ±SD. *p*-values were calculated using a paired t-test. *p*-values <0.05 indicate statistically significant differences between pre- and post-burning measurements.

Supplementary Table S5. Association between behavior level and oxidative stress biomarkers during pre- and post-burning periods

Questions (Awareness) score	N	MDA (nmol/mL) Pre-burning	MDA (nmol/mL) Post-burning	p-value	8-OHdG (pg/mL) Pre-burning	8-OHdG (pg/mL) Post- burning	p-value	1-OHP (µmol/mol creatinine) Pre-burning	1-OHP (µmol/mol creatinine) Post-burning	p-value
Low knowledge (<8)	25	6.72 ± 3.90	6.36 ± 3.75	0.667	47.31 ± 8.93	65.29 ± 8.00	<0.001	0.15 ± 0.21	0.63 ± 0.67	0.001
High knowledge (=9)	114	6.03 ± 3.73	5.93 ± 3.72	0.812	50.39 ± 20.83	64.40 ± 22.86	<0.001	0.24 ± 0.57	0.94 ± 1.36	<0.001

Data are presented as mean ±SD. *p*-values were calculated using a paired t-test. *p*-values <0.05 indicate statistically significant differences between pre- and post-burning measurements.

Supplementary Figure S1. Supplementary Figure 1 shows a STROBE flow diagram of participant recruitment, inclusion, and exclusion criteria.

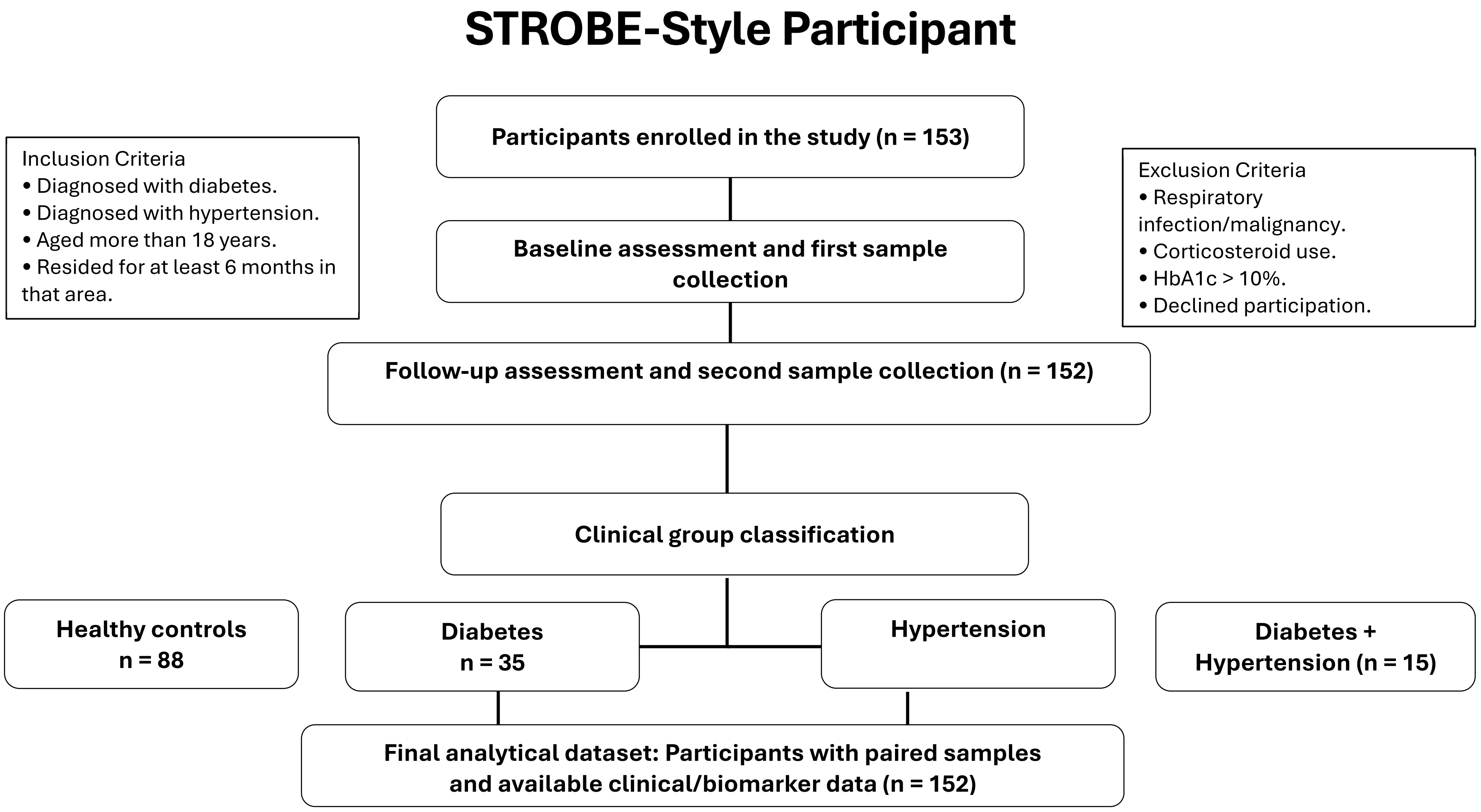

Supplement: Supplementary file 1 [file toxics-14-00771-s001.zip › toxics-4491005-supplementary.pdf]
